# Combining cardiac monitoring with actigraphy aids nocturnal arousal detection during ambulatory sleep assessment in insomnia

**DOI:** 10.1093/sleep/zsac031

**Published:** 2022-03-31

**Authors:** Lara Rösler, Glenn van der Lande, Jeanne Leerssen, Austin G Vandegriffe, Oti Lakbila-Kamal, Jessica C Foster-Dingley, Anne C W Albers, Eus J W van Someren

**Affiliations:** 1 Netherlands Institute for Neuroscience, Department of Sleep and Cognition, Amsterdam, The Netherlands; 2 Department of Integrative Neurophysiology, Center for Neurogenomics and Cognitive Research, Amsterdam Neuroscience, VU University, Amsterdam, The Netherlands; 3 Department of Mathematics and Statistics, Missouri University of Science and Technology, Rolla, MO,USA; 4 Department of Integrative Neurophysiology and Psychiatry, Center for Neurogenomics and Cognitive Research, VU University, Amsterdam UMC, Amsterdam Neuroscience, Amsterdam, The Netherlands

**Keywords:** insomnia, heart rate, heart rate variability, actigraphy, arousal

## Abstract

**Study Objectives:**

The objective assessment of insomnia has remained difficult. Multisensory devices collecting heart rate (HR) and motion are regarded as the future of ambulatory sleep monitoring. Unfortunately, reports on altered average HR or heart rate variability (HRV) during sleep in insomnia are equivocal. Here, we evaluated whether the objective quantification of insomnia improves by assessing *state-related changes* in cardiac measures.

**Methods:**

We recorded electrocardiography, posture, and actigraphy in 33 people without sleep complaints and 158 patients with mild to severe insomnia over 4 d in their home environment. At the microscale, we investigated whether HR changed with proximity to gross (body) and small (wrist) movements at nighttime. At the macroscale, we calculated day-night differences in HR and HRV measures. For both timescales, we tested whether outcome measures were related to insomnia diagnosis and severity.

**Results:**

At the microscale, an increase in HR was often detectable already 60 s prior to as well as following a nocturnal chest, but not wrist, movement. This increase was slightly steeper in insomnia and was associated with insomnia severity, but future EEG recordings are necessary to elucidate whether these changes occur prior to or simultaneously with PSG-indicators of wakefulness. At the macroscale, we found an attenuated cardiac response to sleep in insomnia: patients consistently showed smaller day-night differences in HR and HRV.

**Conclusions:**

Incorporating state-related changes in cardiac features in the ambulatory monitoring of sleep might provide a more sensitive biomarker of insomnia than the use of cardiac activity averages or actigraphy alone.

**Statement of Significance**

It has remained challenging to find objective markers of insomnia. Ambulatory actigraphy assessments frequently fail to detect differences between poor and good sleepers and are not sensitive enough to detect treatment effects. Multisensory wearable devices suggest that the additional monitoring of heart rate might improve sleep-wake classifications, but investigations into heart rate averages during sleep in insomnia remain ambiguous. We examined whether state-related changes in heart rate prior and following nocturnal movements and differences in cardiac measures from day to night might be more informative on insomnia than within-state averages. Our results suggest that state-related changes in insomnia represent a more sensitive biomarker of insomnia severity than the use of actigraphy alone.

## Introduction

Insomnia is the second most prevalent mental disorder and is characterized by difficulties initiating or maintaining sleep [1]. Although sleep complaints often become easily apparent in clinical interviews or in patients’ subjective sleep diaries, obtaining objective markers of insomnia has remained challenging. Polysomnography (PSG) is the gold standard for the objective assessment of sleep, yet PSG findings often show less pronounced differences between patients with insomnia and controls than subjective sleep estimates suggest [2]. Similarly, a recent meta-analysis revealed that sleep estimates obtained from actigraphy are not sufficiently reliable in sleep disorders and frequently fail to detect intervention effects [3].

Neurobiological evidence suggests that chronic hyperarousal is a core feature of insomnia [4, 5]. Accordingly, patients with insomnia may show elevated nocturnal temperature [6], increased sensitivity to auditory stimuli during wakefulness and sleep [7], and restless rapid eye movement (REM) sleep [8]. Chronic hyperarousal should also manifest in autonomic nervous system (ANS) activity, which is commonly assessed by heart rate (HR) and heart rate variability (HRV). Cardiac monitoring could therefore be of value in the development of objective biomarkers of insomnia and its severity.

A recent review revealed, however, that the role of cardiac activity in insomnia remains contested, largely because of small sample sizes, variability in recording lengths, and inconsistent processing techniques across previous studies [9]. Additionally, most studies investigated autonomous activity during night time only, providing incomplete insight into the 24-hour dynamics of the cardiac system in insomnia [10–12].

A more complete view of the 24-hour cardiac dynamics is becoming increasingly feasible due to ongoing technological improvements in its ambulatory assessment with inexpensive wearable devices. Such devices can be used to complement wrist actigraphy, which has become a popular alternative to polysomnography (PSG), given its affordability and relative ease of use [13]. However, common algorithms for analysis of wrist actigraphy data are biased towards the classification of sleep and easily miss wake epochs [14]. While wrist actigraphy trackers have been validated against PSG in the general population [15], their tendency towards overclassification of sleep may be especially problematic for clinical populations. Actigraphy analyses rest on the assumption that prolonged periods of immobility signal sleep. Patients with insomnia, however, may lie still while they are awake, leading to a potential misclassification of epochs without movement as sleep [16, 17]. Concurrent actigraphy and cardiac recordings may help to detect wakefulness in insomnia based on elevated autonomic nervous system activity rather than solely relying on the presence or absence of movement.

In the current study, we examined whether the objective quantification of insomnia can be improved by assessing micro- and macro-scale state-related changes in cardiac measures. To this end, we collected ambulatory electroencephalography (ECG) and actigraphy data from 191 participants in their home environment over a period of four days and nights. At the microscale, time-locked ECG and actigraphy data allowed us to evaluate cardiac changes occurring prior to and following nocturnal postural changes and wrist movements. We hypothesized that alterations in autonomic activity may precede and linger for longer than changes in actigraphy movements and, therefore, provide a more complete view of nocturnal arousals and how these may differ in insomnia. At the macroscale, we investigated whether day-night differences of cardiac measures are more informative on insomnia than the examination of mere daytime or nighttime averages.

## Methods

Participants with insomnia and controls without sleep complaints were recruited through the Netherlands Sleep Registry (www.slaapregister.nl), advertisements, and media to join a longitudinal study on insomnia [18]. We only considered applicants between 18 and 70 years of age. After application, we verified by phone that participants met the inclusion criteria and insomnia criteria when applicable. Exclusion criteria for both participants with insomnia and controls relevant for the current analyses were: a current diagnosis of (1) current major depressive disorder, (2) current treatment with antidepressant medication, (3) current CBT-I treatment, (4) sleep apnea syndrome, moderate to severe restless legs syndrome or severe periodic limb movement disorder, (5) self-reported diagnosis of a severe neurological or psychiatric disorder, (6) self-reported severe physical or mental impairment due to stroke, or traumatic head injury, (7) current shift work (but see [18] for a complete list of exclusion criteria). The use of sleep medication was permitted and monitored (see Supplementary Material and Supplementary Tables S1-S4 for detailed information on sleep medication monitoring and results of nonmedicated patients only). In total, 213 participants were included in the study but due to study drop-out (n = 4), lack of available ECG data (*n* = 13), lack of available sleep diaries (*n* = 7), and issues with data quality (*n* = 5), 184 participants (130 females) were included in the final sample (see Table 1 for further sample characteristics). Of the 184 participants, 152 met the ICSD3 and DSM-5 criteria for Insomnia Disorder. The relatively large number of participants with Insomnia Disorder allowed us to analyze insomnia severity across its entire spectrum rather than merely enforcing a dichotomy between people with and without Insomnia Disorder.

**Table 1.** Sample characteristics

	Insomnia	Controls	*P*
	(*n* = 152)	(*n* = 32)	
Male/Female	45/107	9/23	.999
Age (years)	48.8 (12.5)	48.0 (12.2)	.736
ISI	15.7 (3.64)	2.59 (2.96)	<.001
***Actigraphic sleep estimates***			
Total sleep time (min)	381 (51.0)	396 (44.8)	.096
Sleep efficiency (%)	76% (9.2%)	78% (7.5%)	.056
Wake after sleep onset (min)	109 (43.4)	98 (38.9)	.167
Sleep onset (min)	14.9 (9.2)	11.8 (7.5)	.057

Numbers (%) and mean (standard deviation). Note that actigraphic sleep estimates are based on *n* = 189 recordings (Insomnia *n* = 157; Controls *n* = 32).

### Procedure

Ambulatory recordings were part of a larger study [18] approved by the Medical Ethics Committee of the VU University Medical Centre (NL63139.029.17) and obtained only after participants had provided written informed consent. Participants received wearable sensors during their first lab visit with detailed instructions. For four consecutive days and nights, HR and chest accelerometry was measured in participant’s home environment using movisens EcgMove4 sensors attached to the chest. Additionally, wrist movement was monitored with ActTrust 2 actigraphs (Condor Instruments, São Paulo, Brazil) for nine consecutive days and nights. Participants filled out sleep diaries daily throughout this time period (Consensus Sleep Diary, CSD [19]) to document their nights. As participants differed in how reliably they wore the trackers, the average number of available days per participant was 3.43 (*SD* = 1.19). Prior to the ambulatory assessment, participants filled out multiple questionnaires including a demographics questionnaire and the insomnia severity index (ISI [20, 21]), which were used for the current analyses.

### Data processing

#### Cardiac measures.

We extracted HR in beats per minute, high-frequency HRV (hf-HRV), low-frequency HRV (lf-HRV), lf/hf-HRV, root mean square of successive R-R intervals (RMSSD), and standard deviation of normal-normal (R-R) intervals (SDNN) using the Movisens Data Analyzer software (movisens GmbH, Karlsruhe, Germany) from the raw ECG signal. The software detects R-peaks with an adaptation of the algorithm of Hamilton et al. (2002) [22] and subsequently uses a method proposed by Clifford et al. (2002) [23] to dismiss all peaks that do not stem from normal heartbeats. Mean values of HR were outputted in 30 s intervals. HRV variables were calculated over segments of 2 min duration. Finally, we calculated normalized hf-HRV (HFnu) as hf-HRV divided by the sum of lf-HRV and hf-HRV. Since normalized lf- and hf-HRV are no independent constructs [24], and since the meaning and proper interpretation of lf-HRV is debated, we made an a priori decision to only consider HFnu as a frequency-domain HRV signal in our analyses. Hfnu as well as RMSSD are believed to reflect parasympathetic activity, whereas SDNN represents total HRV [25].

### Postural changes

Chest accelerometry was processed using a thresholded stream clustering algorithm. Using the y-axis (shoulder-to-shoulder) and z-axis (through-the-chest) channels, a distance of epsilon = 1.025 between two consecutive vectors signaled a posture shift. Of the detected stable postures, clusters that were less than 30 s in length were merged forward with the next cluster to reduce the number of clusters and maintain a minimum of a 30-s epoch. The x-axis (head-to-toe channel) did not contain significant amounts of movement and was not considered when obtaining stable postures (see Supplementary Material for a more detailed explanation of the algorithm).

To investigate changes in HR *prior* to a movement, we subsequently read out all nocturnal postural changes that occurred after at least 10 min of postural immobility. This was done to ensure that potential increases in HR are not influenced by prior proximal arousals. Since not all participants had instances that fulfilled this criterion, data of 164 participants (135 patients with insomnia and 29 controls) were included in the subsequent analyses. The proportions of included participants was not different for people with insomnia and controls (Chi-square = 0.133, *p* = .715). On average, postural changes preceded by at least 10 minutes of immobility occurred 13.08 (*SD* = 6.70) times per person per night. Participants with insomnia and controls did not differ in their number of postural changes (*t* = –0.57, *p* = .572).

Similarly, to examine changes in HR *following* a movement, we selected all nocturnal postural changes which were not followed by another postural change for at least 10 minutes to avoid subsequent influences on HR. 165 participants (136 patients with insomnia and 29 controls) had instances that fulfilled this criterion and contributed data to the analyses. On average, postural changes followed by at least 10 minutes of immobility occurred 15.45 (*SD* = 8.86) times per person per night and their frequency did not differ between patients and controls (*t* = –0.83, *p* = .410).

### Wrist movements

Generally, raw wrist movements were much more abundant than postural changes detected by chest accelerometry. To cancel out miniscule movements that might not be representative of nocturnal arousal, we decided to first derive epoch-wise sleep versus wake estimates to differentiate between moments of no or little movement from moments of high wrist movement. We obtained epoch-wise sleep versus wake estimates using the open-source package *PyActigraphy* [26] implementing a device-tuned version of the Cole-Kripke algorithm provided by actigraph manufacturer Condor Instruments. Epochs were analyzed within sleep diary-derived bedtimes and get-up times. Paralleling the postural change analysis, we first read out all high wrist movement episodes that occurred after a minimum of at least 10 minutes of no or little wrist movement. This analysis included data of 131 patients with insomnia and 30 controls. On average, participants had 10.84 (*SD* = 4.30) episodes of wrist movements classified as wakefulness following 10 minutes of no to little movement per night. Again, participants with insomnia and controls showed no differences in the number of these small movements (*t* = 0.09, *p* = .923). HR changes following wrist movement were evaluated by selecting all high wrist movement episodes which were followed by 10 minutes of no wrist movement classified as wakefulness. Such episodes were found in 131 patients with insomnia and 29 controls. On average, they had 11.51 (*SD* = 4.77) of these episodes per night, which did not differ between patients and controls (*t* = 0.29, *p* = .773).

### Statistical analyses

At the microscale, we investigated how HR changed prior to and following nocturnal movements. We refrained from examining microscale changes in HRV variables as reliable HRV assessments require integrating at least two minutes of data, which is not adequate for our analysis at 30 s increments. For both postural changes and wrist movements, we used two separate linear mixed models implemented in the R-package *lme4* [27]. Mixed effect models allow for a variable number of episodes within a variable number of nights per subject. The models evaluated (1) changes in HR prior to an impending postural change and (2) changes in HR following a previous postural change. These two models were each run twice: first regarding insomnia as a dichotomy, i.e., present or absent, and subsequently regarding insomnia as a continuum using the ISI score, to allow for inferences across the entire spectrum of insomnia severity. Accordingly, regression models first evaluated the dependency of HR on the factorial predictor “distance from movement” (intervals of 30 s at either 90, 60, or 30 s either prior or following the movement which were chosen after data visualization, see below), the dichotomous predictor insomnia diagnosis, and their interaction. Subsequently, these analyses were repeated using the continuous variable insomnia severity instead of dichotomous. Age and gender were included as covariates.

Pairwise comparisons per 30 s interval were calculated using the R-package *emmeans,* which uses the Tukey adjustment to correct for multiple comparisons. We then investigated whether these differences in HR across distance from movement differed as a function of insomnia by implementing contrasts. Specifically, we were interested in whether the stepwise differences in HR between 90-60, 60-30, and 30-0 s before movement were associated with insomnia diagnosis or severity.

At the macroscale, we investigated sleep-wake state-dependent differences in HR and HRV indices. Wakefulness and sleep windows were defined from sleep diaries. Mixed effect models allow for a variable number of 30-s (for HR) or 2-minute (for HRV) episodes within a variable number of nights and days per subject. The models first evaluated the dependency of HR or HRV measures (HFnu, Sdnn, Rmssd) on the binary regressor sleep versus wake, the dichotomous predictor insomnia group, and their interaction. Paralleling the microscale analyses, we re-ran this model using insomnia severity as a continuous measure instead of dichotomous. We again included subject identity as a random intercept and included age and gender as covariates in both of these models.

We refrained from standardizing our variables so that coefficient estimates can be more meaningfully interpreted. Significance of predictors was estimated with an analysis of variance table using Satterwaithe’s method in the R-package *lmerTest* [28]. We considered an effect significant if the *p*-value was < .05.

## Results

### Heart rate increases prior to nocturnal postural changes

**Fig. 1.**
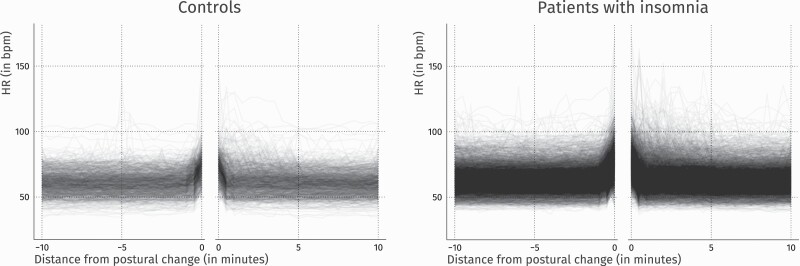
Heart rate prior and after a nocturnal postural change. Lines indicate individual trajectories to and from single movement episodes. The missing central part indicates movement episodes of differing length.

Figure 1 visualizes changes in HR as they occur at the microscale before and after a nocturnal movement. Each line shows the trajectory to and from an individual movement episode. The left side shows HR trajectories starting 10 minutes prior to a postural change until the epoch containing the onset of the postural change. The right side shows an individual participant’s average HR trajectory starting from the last epoch containing postural change up until 10 minutes afterwards. The plots do not show the variable number of epochs that contain postural change in between.

As the plot revealed a clear increase of HR just prior to postural changes, analyses focused on the three 30 s epochs preceding the movement. Mixed model analyses revealed significant main effects: HR changed with distance from the postural change (F_(3, 22489)_ = 1714.17, *p* =< .001) and was consistently higher in participants with a diagnosis of insomnia (F_(1, 157)_ = 6.91, *p* = .009). Moreover, interaction effects indicated a relatively higher HR in participants with insomnia than in controls in the two epochs before the onset of a postural change (F_(3, 22489)_ = 4.16, *p* = .006) (Table 2, upper part).

**Table 2.** Heart rate levels in the sequential 30 s epochs preceding the epoch containing the onset of a postural change (upper part), as well as changes in heart rate between subsequent epochs (lower part)

Interval (seconds before epoch with postural change onset)	Insomnia	Controls	Group differences	
	HR average (bpm)	HR average (bpm)	HR average (bpm)	*P*
90–60	63.1	59.4	3.77	.016
	[61.70, 64.50]	[56.51, 62.29]	[0.69, 6.85]	
60–30	63.4	59.8	3.58	.023
	[62.00, 64.80]	[56.90, 62.69]	[0.50, 6.66]	
30–0	67.0	62.7	4.35	.006
	[65.60, 68.40]	[59.81. 65.59]	[1.27, 7.42]	
Epoch with postural change onset	75.0	70.3	4.70	.002
	[73.60, 76.40]	[67.41, 73.19]	[1.62, 7.78]	
	Insomnia	Controls	Group differences	
**Interval contrast** (seconds before epoch with postural change onset)	**HR change (bpm)**	HR change (bpm)	HR change (bpm)	P
90–60 to 60–30	0.25	0.43	0.18	.605
	[–0.05, 0.55]	[–0.21, 1.07]	[–0.52, 0.89]	
60–30 to 30–0*	3.67	2.91	–0.77	.033
	[3.38, 3.98]	[2.27, 3.55]	[–1.48, –0.06]	
30–0 to epoch with postural change onset*	7.91	7.56	–0.36	.325
	[7.61, 8.21]	[6.92, 8.20]	[–1.06, 0.35]	

Asterisks indicate a systematic change in HR, i.e. differing significantly from zero. Confidence intervals are included in square brackets.

The lower part of Table 2 shows the group averages of between-epoch differences. Mixed effect analyses revealed main effects: across groups, HR increased significantly from 60–30 to 30–0 s before the epoch containing the onset of the postural change, and again significantly from 30–0 s to the epoch containing the onset of the postural change. Moreover, an interaction effect indicated that people with insomnia showed a stronger increase in HR from 60-30 sec to 30-0 sec prior to the epoch containing the onset of the postural change (respectively 3.67 bpm ± 0.15 in insomnia and 2.91 ± 0.33 in controls, *t* = 2.14, *p* = .033).

**Fig. 2.**
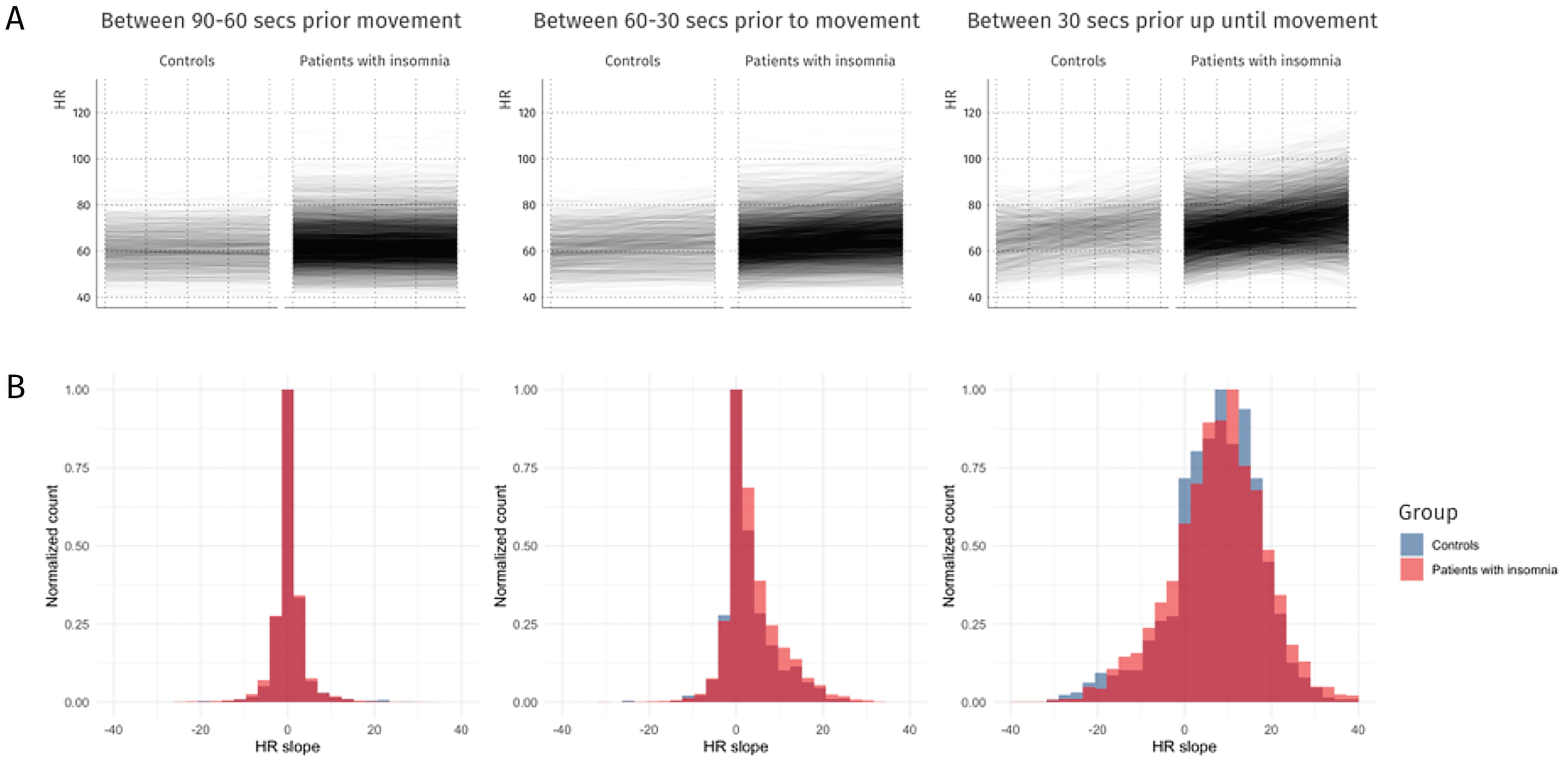
Relationship between between-epoch HR differences and insomnia diagnosis prior to nocturnal postural change. A) Individual HR differences during the three 30 s epochs prior to nocturnal postural change. B) Normalized histograms of HR differences per epoch.

After investigating group differences, we examined whether HR trajectories prior to a postural change were associated with insomnia severity as a continuous variable. While there was no significant main effect of insomnia severity on HR across this 90 s window (F_(1, 156)_ = 2.15, *p* = .145), there was a significant interaction effect between insomnia severity and distance from postural change on HR (F_(3, 22358)_ = 9.97, *p* =< .001). To better understand what is driving this effect, we redefined the default contrasts to examine whether insomnia severity differently affected stepwise differences in HR from 90–60, 60–30, and 30–0 s before movement. Subsequent analyses on HR changes between epochs revealed that insomnia severity was associated with the steepness of the increase in HR that occurred between 60–30 and 30–0 s prior to the epoch containing the onset of the postural change. For every single point increase in insomnia severity, HR increased by 0.44 (see Table 3 and Figure 2).

**Table 3.** Estimates of the effect of insomnia severity on changes in heart rate between subsequent epochs before the onset of a postural change

Interval (seconds)	Insomnia severity	*P*
	HR change (bpm)	
Epoch with offset of postural change to 0–30	–0.35	.020
	[–0.65, –0.06]	
0–30 vs 30–60	0.06	.694
	[–0.24, 0.36]	
30–60 vs 60–90	–0.02	.913
	[–0.32, 0.28]	

### Continued elevated heart rate following nocturnal postural changes

To examine whether decreases in HR following a nocturnal postural change are altered in insomnia, we repeated the above analysis, but now investigating the 90 s following a postural change. Mixed model analyses again revealed significant main effects: HR changed with distance from the termination of postural change (F_(3, 32186)_ = 996.70, *p* =< .001), and was consistently higher in participants with a diagnosis of insomnia (F_(1, 160)_ = 6.77, *p* = .010, see Table 4 for HR averages and changes in patients and controls). Unlike the HR before a pending postural change, there was no significant interaction effect between insomnia diagnosis and distance after postural change (F_(3, 175)_ = 1.88, *p* = .130).

**Table 4.** Heart rate levels in the sequential 30 s epochs following the epoch containing the offset of a postural change (upper part), as well as changes in heart rate between subsequent epochs (lower part)

Interval (seconds)	Insomnia	Controls	Group differences	
	HR average	HR average	HR average	
	(bpm)	(bpm)	(bpm)	*P*
Epoch with offset of postural change	74.8	70.0	4.73	.003
	[73.38, 76.21]	[67.07, 72.93]	[1.57, 7.89]	
0–30	66.6	62.6	4.01	.013
	[65.18, 68.02]	[59.67, 65.52]	[0.85, 7.16]	
30–60	65.1	61.2	3.87	.017
	[63.68, 66.51]	[58.27, 64.12]	[0.71, 7.03]	
60–90	65.0	61.0	4.02	.013
	[63.58, 66.42]	[58.07, 63.92]	[0.86, 7.18]	
	Insomnia	Controls	Group differences	
**Interval contrast** **(seconds)**	HR change	HR change	HR change	
	(bpm)	(bpm)	(bpm)	*P*
Epoch with offset of postural changeto 0–30*	–8.14	–7.41	0.72	.072
	[–8.46, –7.81]	[–8.12, –6.69]	[–0.06, 1.50]	
0–30 vs 30–60*	–1.52	–1.38	0.14	.724
	[–1.85, –1.19]	[–2.09, –0.67]	[–0.64, 0.92]	
30–60 vs 60–90	0.08	0.24	0.15	.325
	[–0.41, 0.25]	[–0.95, 0.47]	[–0.63, 0.93]	

Asterisks indicate a systematic change in HR, i.e. differing significantly from zero.

We subsequently examined whether changes in HR following a nocturnal postural change were associated with insomnia severity. The relationship between insomnia severity and HR in this 90 s window was only trend-level significant (F_(1, 158)_ = 3.67, *p* = .057), as was the interaction effect with distance from postural change on HR (F_(3, 32015)_ = 2.25, *p* = .081). Table 5 shows that a 1-point increase in insomnia severity was associated with 0.35 stronger drop in the initial HR decline immediately following a movement.

**Table 5.** Estimates of the effect of insomnia severity on changes in heart rate between subsequent epochs following the offset of a postural change

Interval (seconds)	Insomnia severity	*P*
	HR change (bpm)	
Epoch with offset of postural change to 0–30	–0.35	.020
	[–0.65, –0.06]	
0–30 vs 30–60	0.06	.694
	[–0.24, 0.36]	
30–60 vs 60–90	–0.02	.913
	[–0.32, 0.28]	

### Heart rate increases prior to nocturnal wrist movement

To investigate whether the observed changes in HR were restricted to postural changes as detected by chest accelerometry, we next repeated the above analyses for wrist movements. Visual inspection of HR prior and following increased wrist movement, classified by the Cole-Kripke algorithm, showed that changes in HR are again limited to a short period prior and following movement (see Supplemental Figure S2). Mixed model analyses revealed only one significant main effect: HR changed with distance from the epoch with wrist movement (F_(3, 15768)_ = 140.76, *p* = < .001), but was only marginally higher in participants with a diagnosis of insomnia (F_(1, 157)_ = 4.62, *p* = .0*5*5). There was no interaction effect between distance from wrist movement and insomnia group (F_(3, 15768)_ = 0.44, *p* = .718) (see Supplemental Table S5, upper part). Across groups, HR increased significantly from 30–0 s to the epoch containing the onset of the wrist movement. However, the between-epoch differences in HR were not significantly different between patients and controls at any timepoint (all *p* > .356, see Supplemental Table S5).

We subsequently investigated whether insomnia severity instead of insomnia diagnosis would reveal a relationship between with HR trajectories prior to a nocturnal wrist movement. However, we again failed to observe a significant main effect of insomnia severity in this 90 s window (F_(1, 155)_ = 1.27, *p* = .261), nor was there an interaction effect between insomnia severity and distance from wrist movement on HR (F_(3, 15666)_ = 0.79, *p* = .498).

### Heart rate decreases following nocturnal wrist movement

To examine whether decreases in HR following a nocturnal wrist movement differ between patients with insomnia and controls, we performed a linear mixed model investigating the 90 s following the movement. Mixed model analyses again revealed only one significant main effect: HR changed with distance from the termination of the wrist movement (F_(3, 16178)_ = 7.12, *p* =< .001), but was only marginally higher in participants with a diagnosis of insomnia (F_(1, 156)_ = 2.86, *p* = .093, see Supplemental Table S6 for HR averages and changes in patients and controls). There was no significant interaction effect between insomnia diagnosis and distance from the last epoch with wrist movement (F_(3, 16178)_ = 0.49, *p* = .689).

When investigating insomnia severity instead of the dichotomous variable insomnia diagnosis, we also failed to observe a general effect of insomnia severity on HR following a wrist movement (F_(3, 16178)_ = 7.12, *p* = <.001) or an interaction effect between insomnia severity and distance from wrist movement (F_(3, 16067)_ = 0.20, *p* = .894).

### Insomnia is associated with smaller day-night differences in cardiac measures

**Fig. 3.**
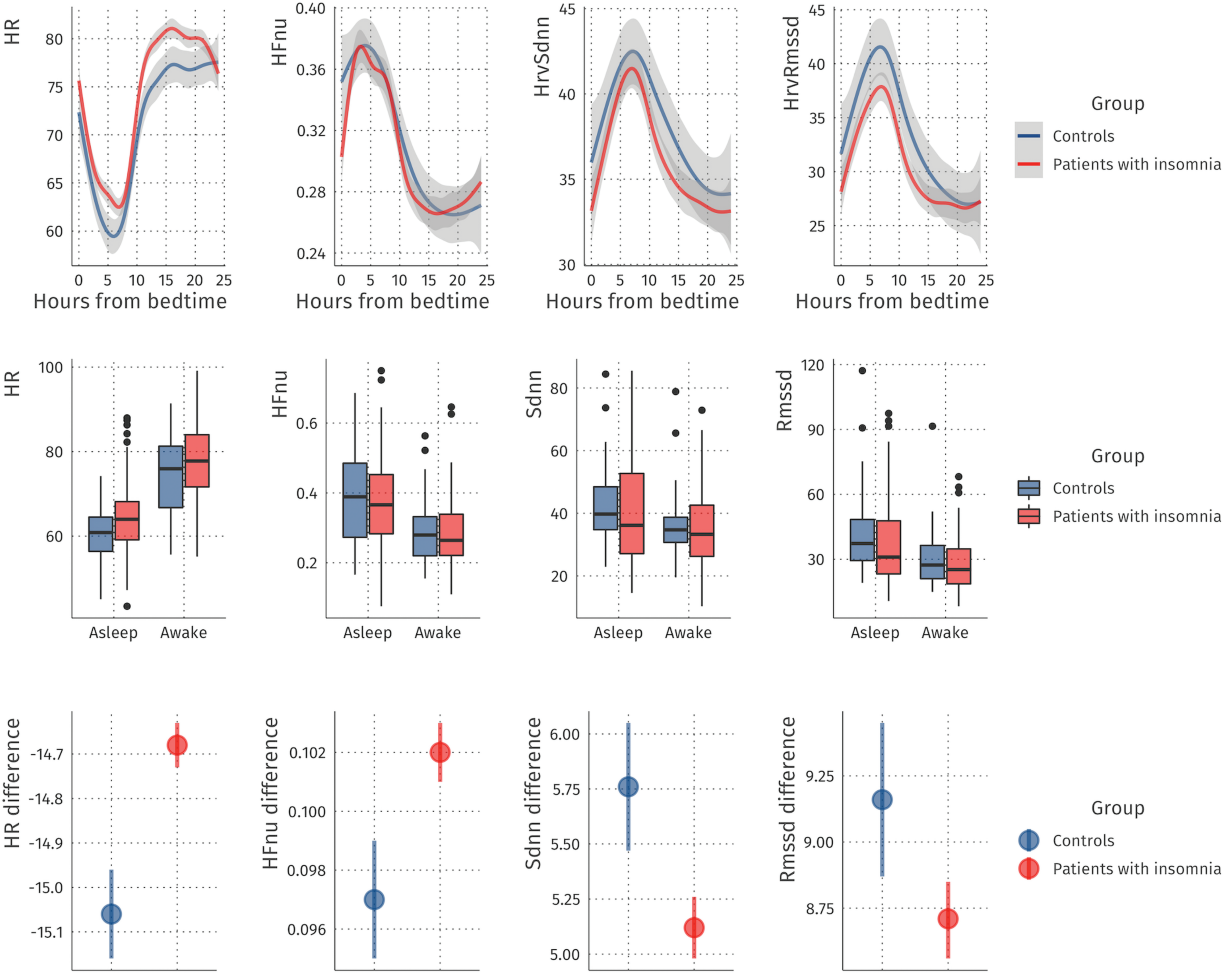
Cardiac measures across day and night. The upper panel shows heart rate, normalized high frequency heart rate variability, the standard deviation between normal-normal heartbeats, and the root mean square of the successive differences between heartbeats separately for patients with insomnia and controls as a function of time from bedtime. Values of multiple days were first aggregated per subject for corresponding times of the day and then averaged across subjects. The middle panels show these measures averaged within daytime wake and nocturnal sleep windows. The lower panels show the behavioral state-dependent differences

We next investigated at the macroscale whether sleep-wake differences of cardiac measures are more informative on insomnia than the examination of mere daytime wake or nighttime sleep averages. Sleep diaries were used to classify epochs as belonging to a time window of daytime wakefulness or to a time window of nocturnal sleep. We used each cardiac measure as an outcome in a mixed model to investigate whether behavioral state-dependent differences distinguish insomniacs from controls and whether behavioral state-dependent differences are associated with insomnia severity as a continuous variable. Indeed, these analyses revealed an interaction effect between insomnia diagnosis and the behavioral state (awake vs asleep) for HR (β _Group*State_ = –0.39, *p <* .001), SDNN (β _Group*State_ = 0.65, *p <* .001), RMSSD (β _Group*State_ = 0.45, *p =* .007), as well as HFnu (β _Group*State_ = 0.005, *p =* .001, see Figure 3 and Table 6 for an overview of these differences, as well as Supplementary Table S7 and S4 for full model results and results of sensitivity analyses that excluded patients taking sleep medication. When thus limiting the sample size, interaction effects between insomnia diagnosis and state no longer reached significance for SDNN and RMSSD). Investigations of insomnia severity as a continuous measure likewise revealed significant interactions with the behavioral state (awake vs asleep) for all cardiac outcome variables. More severe insomnia symptoms were associated with smaller differences between sleep versus wake states in HR (β _ISI*State_ = -0.05, *p =* .022), HFnu (β _ISI*State_ = 0.002, *p <* .001), SDNN (β _ISI*State_ = 0.73, *p <* .001) and RMSSD (β _ISI*State_ = 1.12, *p <* .001).

**Table 6.** Differences in cardiac measures during sleep windows relative to wake windows for people with insomnia and controls.

	Insomnia	Controls	Group differences	
				*P*
*HR*_*sleep-wake*_	–14.68	–15.06	–0.385	<.001
	[–14.73, –14.63]	[–15.16, –14.96]	[–0.50, –0.27]	
*HFnu*_*sleep-wake*_	0.102	0.097	0.005	.001
	[0.101, 0.103]	[0.095, 0.099]	[0.001, 0.009]	
*SDNN*_*sleep-wake*_	5.12	5.76	0.645	<.001
	[4.98, 5.26]	[5.47, 6.05]	[0.32, 0.97]	
*RMSSD*_*sleep-wake*_	8.71	9.16	0.452	.007
	[8.56, 8.85]	[8.87, 9.45]	[0.13, 0.77]	

## Discussion

The current investigation evaluated whether objective ambulatory quantification of insomnia may improve by assessing micro- and macro-scale state-related changes in cardiac measures. To do so, we used ambulatory assessments obtained in the home environment. At the microscale, we observed that altered cardiac activity precedes and follows nocturnal movements. Already a minute prior to wrist movement and postural changes, heart rate significantly increased, thereby representing an earlier indicator of nocturnal arousal than the movement itself. The increase in heart rate prior to postural changes was more pronounced for participants with more severe insomnia. This finding suggests that nocturnal monitoring of cardiac activity along with postural changes may benefit quantification of insomnia severity. Moreover, at the macroscale, we observed smaller behavioral state-related differences in cardiac activity in participants with more severe insomnia, indicating that 24-hr cardiac activity monitoring can improve objective quantification of insomnia severity.

### Changes in heart rate precede and follow nocturnal movements

HR and HRV are known to follow a circadian rhythm, with a decrease of HR and an increase of HRV during the night [29, 30]. With respect to nocturnal gross movements, we observed a clear increase in HR starting 60 s prior to a postural change and a significant decrease in HR up until 60 s following the termination of the postural change. Similarly, wrist movements were also preceded and followed by changes in HR up to 30 s prior and after their occurrence. Previous research pointed to an increase in heart rate about 10–20 beats prior to an EEG arousal [31], which is in line with our observation. Considering the lack of simultaneous PSG recordings, it is, however, not possible for us to discern whether these increases in heart rate precede actual awakenings or whether they are coincident with wakefulness without movement yet. These findings nonetheless demonstrate why actigraphy alone might struggle with relatively low specificity for wake episodes [32], because arousal visible in modalities other than motion remains undetected. Independent of whether cardiac changes constitute wake episodes or occur just prior to wake episodes, the observed increases in sympathetic activation can aid our understanding of how restful or restless a night’s sleep was. It is even conceivable that the overestimation of subjective wakefulness experienced by people with insomnia reflects a state with sleep EEG still indicating sleep while cardiac measures suggest wakefulness. Current commercially available wearable sleep sensors (e.g. Apple Watch, Oura Ring, and Fitbit devices) now increasingly combine the measurement of motion and heart rate for their behavioral state estimates. While these consumer-level wearables often lack available technical disclosure of the algorithms used [33], other researchers have shown that the addition of cardiac measures to actigraphy leads to better sleep classification results in people without sleep complaints [34, 35]. Our findings shed further light into mechanisms underlying this improved sleep prediction by demonstrating that cardiac changes start earlier and last longer than movement episodes themselves.

### Cardiac monitoring aids the assessment of insomnia

Crucially, we observed a steeper increase in heart rate prior to a postural change in participants with higher insomnia severity. This difference in heart rate slope was restricted to the onset of the rise that was first systematically present in the interval of 60–30 s prior to the movement. Patients with insomnia also show a longer continuation of an elevated heart rate following a postural change. Previous studies showed that nocturnal awakenings, although not more frequent, are prolonged in insomnia [36, 37]. The continued elevation in heart rate might therefore represent a continued awakening, or, alternatively, increased sympathetic activation that continues into sleep. Although actigraphy algorithms have been fine-tuned to work better for insomnia [38], actigraphy alone currently does not suffice to properly distinguish insomnia from good sleep [13]. Such discrimination is essential because insomnia is becoming a societal problem of much higher prevalence than insufficient sleep duration [39]. Based on the current findings, nocturnal monitoring of cardiac activity alongside movement may help to improve the utility of ambulatory sleep assessments for insomnia.

### Continuous cardiac hyperarousal in insomnia

Next to the nocturnal state-related changes in cardiac activity on the microscale, we also investigated at the macroscale whether behavioral state-dependent differences are more sensitive to distinguish people with insomnia from controls and are associated with insomnia severity as a continuous variable. The results indicate smaller state-dependent differences in patients with insomnia than in controls. Moreover, the state-dependent differences become increasingly smaller with increasing insomnia severity. Typically, the autonomic nervous system shifts from sympathetic predominance characteristic of wakefulness towards parasympathetic predominance during non-REM sleep [40]. Smaller decreases in wake-to-sleep HR and smaller increases in wake-to-sleep temporal-domain HRV (in particular RMSSD which is thought to reflect parasympathetic activity) in insomnia indicate that this shift from sympathetic towards parasympathetic dominance is less successful in people with insomnia. Overall, the observed attenuation of differences in cardiac activity in the transition between day and night thus extends the idea of continuous, “round-the-clock” hyperarousal in insomnia. Rather than overall elevated arousal levels in insomnia, we found a smaller reduction of arousal from day to night in insomnia. Previous work indicated that even during deep (N3) sleep, people with insomnia show EEG activity that is representative for wakefulness and shallow sleep [41–43]. In contrast to the substantial number of reports on wake-like EEG features during sleep in insomnia, only one study reported an attenuated drop in HR in the transition from wake to sleep in people with insomnia [12]. This previous finding and our 24-hr results concertedly support the importance of monitoring cardiac indicators of hyperarousal across wake and sleep.

### Limitations and future directions

Our goal was to assess the monitoring of insomnia severity and symptoms in the home environment using mobile sensors. As we did not include ambulatory PSG recordings, one major limitation of our study is that we cannot determine whether the increased heart rate occurring prior movement indicates sympathetic arousal taking place while still asleep or coinciding with wakefulness without movement. Irrespective of whether the observed steeper increase and flatter decrease of heart rate in insomnia signal a wake period, these differences between patients with insomnia and controls can still provide valuable information to the puzzle of poor subjective sleep in insomnia. Nonetheless, future research conducting PSG recordings alongside actigraphy and ECG is necessary to clarify whether these changes in heart rate occur prior to or simultaneously with awakenings.

It additionally needs to be noted that ambulatory data are also commonly noisier than data acquired in the lab. Standard deviations for day-night differences in cardiac measures were quite high and possibly arose because of imprecision in the subjective retrospective definition of sleep and wake times or general noise in the measurements. Efforts to replace subjective bedtime estimates with objective assessments (e.g. light, pressure, or presence sensors), may help to reduce measurement error and thus optimize the precision of the obtained features. Frequency-domain HRV results also stand out in our study, as these are the only measure in which patients show a steeper change from day to nighttime—although differences are miniscule. Future research is necessary to investigate whether this observation is affected by measurement error or whether frequency-domain HRV truly shows increased state-related changes in insomnia. Temporal-domain HRV differences also did not hold when medicated patients were excluded from the analysis, despite similar levels of insomnia severity in this subgroup. Again, future research is needed to determine whether the observed deviations in results truly reflect medication effects or are due to a loss of power by reducing the sample size. Overall, developments in mobile heart rate recordings, including pulse volume measures, are ongoing and assessments become more reliable [44]. We currently provide a proof of principle, showing that developments for better characterization of sleep quality would benefit from the combination of movement and cardiac activity recordings. The synchronized investigation of the development of both heart rate and activity over time thus provides a promising avenue for future research. Crucially, ambulatory recordings require detailed investigations and controls of signal quality and exclusion of outliers is vital to advance ambulatory sleep monitoring in insomnia.

## Conclusion

In conclusion, we observed a clear relationship between cardiac activity and nocturnal movement in the current study. Heart rate started to increase 60 s prior to a gross or small nocturnal movement, thereby signaling a nocturnal arousal earlier than the movement itself. A continued increase in heart rate could additionally be detected up until 60 s longer than the movement, suggesting that cardiac activity may provide more detailed insights into nocturnal arousals than movements alone. The increase in heart rate was more pronounced in participants with insomnia prior to postural changes, but not prior to small movements. Additionally, sleep versus wake differences in cardiac measures were smaller in people with insomnia and more attenuated with increasing insomnia severity. Taken together, our results suggest that the inclusion of cardiac and postural measures in ambulatory devices to estimate sleep might provide a more sensitive biomarker of insomnia severity than the exclusive use of actigraphy.

## Supplementary Material

zsac031_suppl_Supplementary_Material
