# Supplementary material for: Combining cardiac monitoring with actigraphy aids nocturnal arousal detection during ambulatory sleep assessment in insomnia
Source: Sleep. 2022 Mar 31;45(5):zsac031. doi: 10.1093/sleep/zsac031 (PMC9113014; doi:10.1093/sleep/zsac031)
Supplement: zsac031_suppl_Supplementary_Material [file zsac031_suppl_supplementary_material.docx]

**Supplementary Material of:**

**Combining cardiac monitoring with actigraphy aids nocturnal arousal detection during ambulatory sleep assessment in insomnia**

Lara Rösler^1^, Glenn van der Lande^1^, Jeanne Leerssen^1,2^, Austin G Vandegriffe^3^, Oti Lakbila-Kamal^1^, Jessica C Foster-Dingley^1^, Anne Albers^1^, Eus van Someren^1,4^

^1^Netherlands Institute for Neuroscience, Department of Sleep and Cognition

^2^Departments of Integrative Neurophysiology, Center for Neurogenomics and Cognitive Research, Amsterdam Neuroscience, VU University, Amsterdam, The Netherlands

^3^Missouri University of Science and Technology, Rolla, MO, USA

^4^Departments of Integrative Neurophysiology and Psychiatry, Center for Neurogenomics and Cognitive Research, VU University, Amsterdam UMC, Amsterdam Neuroscience, Amsterdam, The Netherlands

Corresponding author: Lara Rösler

l.rosler@nin.knaw.nl

**Medication details and results including non-medicated patients only**

Of the 184 patients entering the overall heart rate variability analyses, 21 patients were taking prescription hypnotics (temazepam, diazepam, lorazepam, oxazepam, zolpidem or zopiclon). Of the 164 participants entering the postural change analysis, 17 patients with insomnia were taking hypnotics. For the postural change analysis, none of the reported significant effects were altered when these subjects were excluded from the respective analyses. However, in the 24-hr analysis evaluating day-night changes in heart rate and HRV, temporal-domain HRV analyses no longer show an interaction effect between insomnia diagnosis and state when the medicated participants are dropped. Notably ISI scores do not differ between medicated and non-medicated patients (*t* = 0.13 , *p* = 0.893), suggesting that changes in prior observed effects cannot be explained by changes in insomnia severity in the sample. Below are result tables of our reported main effects, when only non-medicated patients with insomnia are included.

|  | **Insomnia**  **(non-medicated)** | **Controls** | **Group differences** | |
| --- | --- | --- | --- | --- |
| **Interval**  (seconds before epoch with postural change onset) | **HR average (bpm)** | **HR average (bpm)** | **HR average (bpm)** | ***p*** |
| 90–60 | 62.9  [61.39, 64.40] | 59.3  [56.40, 62.19] | 3.62  [0.46, 6.78] | .025 |
| 60–30 | 63.2  [61.69, 64.70] | 59.7  [56.80, 62.59] | 3.44  [0.28, 6.59] | .032 |
| 30–0 | 66.9  [65.39, 68.40] | 62.6  [59.71. 65.49] | 4.30  [1.44, 7.46] | .008 |
| Epoch with postural change onset | 74.7  [73.19, 76.20] | 70.2  [67.31, 73.09] | 4.49  [1.33, 7.64] | .005 |
|  |  |  |  | |
|  | **Insomnia**  **(non-medicated)** | **Controls** | **Group differences** | |
| **Interval contrast**  (seconds before epoch with postural change onset) | **HR change (bpm)** | **HR change (bpm)** | **HR change (bpm)** | ***p*** |
| 90–60 to 60–30 | 0.26  [-0.06, 0.58] | 0.43  [-0.21, 1.07] | 0.18  [-0.54, 0.90] | .629 |
| 60–30 to 30–0* | 3.76  [3.44, 4.08] | 2.91  [2.27, 3.55] | -0.85  [-1.56, -0.13] | .020 |
| 30–0 to epoch with postural change onset* | 7.75  [7.42, 8.07] | 7.56  [6.92, 8.20] | -0.36  [-1.07, 0.36] | .598 |

**Table S1. Heart rate levels in the sequential 30 seconds epochs preceding the epoch containing the onset of a postural change (upper part), as well as changes in heart rate between subsequent epochs (lower part).** Asterisks indicate a systematic change in HR, i.e. differing significantly from zero. Confidence intervals are included in square brackets.

|  | **Insomnia severity** | |
| --- | --- | --- |
| **Interval contrast**  (seconds before epoch  with postural change onset) | **HR change (bpm)** | ***p*** |
| 90–60 to 60–30 | -0.062  [-0.35, 0.23] | .672 |
| 60–30 to 30–0 | 0.484  [0.20, 0.77] | .001 |
| 30–0 to epoch with  postural change onset | 0.046  [-0.24, 0.33] | .756 |

**Table S2. Estimates of the effect of non-medicated insomnia severity on changes in heart rate between subsequent epochs before the onset of a postural change.**

|  | **Insomnia severity** |  |
| --- | --- | --- |
| **Interval**  **(seconds)** | **HR change**  **(bpm)** | ***p*** |
| Epoch with offset of  postural change to 0–30 | -0.24  [-0.56, 0.07] | .128 |
| 0–30 vs 30–60 | 0.02  [-0.29, 0.34] | .559 |
| 30–60 vs 60–90 | -0.05  [-0.37, 0.27] | .809 |

**Table S3. Estimates of the effect of non-medicated insomnia severity on changes in heart rate between subsequent epochs following the offset of a postural change.**

|  | **Insomnia** | **Controls** | **Group differences** | |
| --- | --- | --- | --- | --- |
|  |  |  |  | ***p*** |
| *HR_sleep-wake_* | -14.79  [-14.84, -14.74] | -15.06  [-15.16, -14.96] | -0.273  [-0.39, -0.16] | <.001 |
| *HFnu_sleep-wake_* | 0.102  [0.100, 0.104] | 0.098  [0.096, 0.100] | 0.004  [0.000, 0.008] | .019 |
| *SDNN_sleep-wake_* | 5.54  [5.39, 5.69] | 5.82  [5.53, 6.11] | 0.278  [-0.05, 0.61] | .099 |
| *RMSSD_sleep-wake_* | 9.27  [9.12, 9.42] | 9.18  [8.88, 9.48] | 0.095  [-0.24, 0.43] | .578 |

**Table S4. Differences in cardiac measures during sleep windows relative to wake windows for non-medicated people with insomnia and controls.**

**Stable Posture Identification Algorithm**

The stable posture identification algorithm is a single linkage clustering through time. If two consecutive points are too far apart, subject to a user defined threshold, then the link is broken and a new cluster is started. We define a stable posture to be a posture which persists for a least 30 seconds. Postures which do not meet this criterion are merged with the subsequent posture to maintain a 30-second epoch and to decrease the number of detected postures.


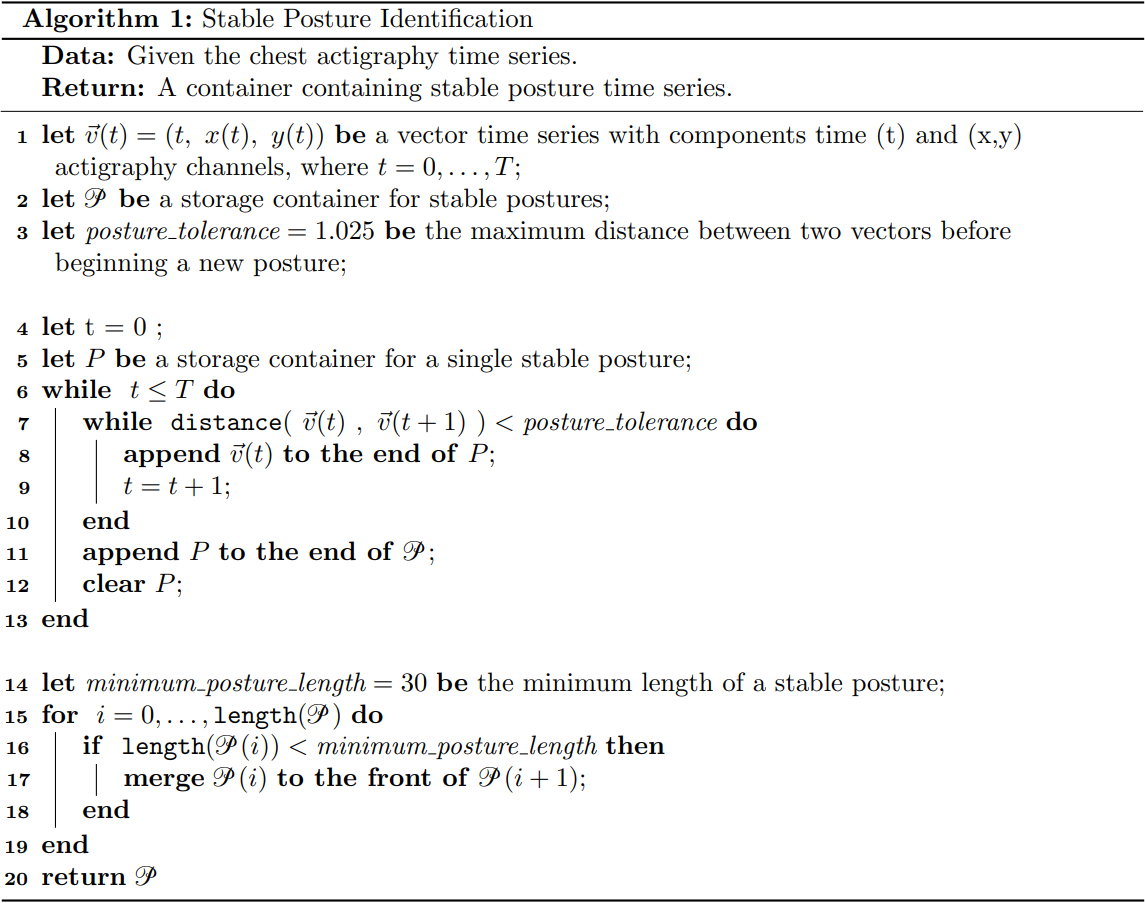


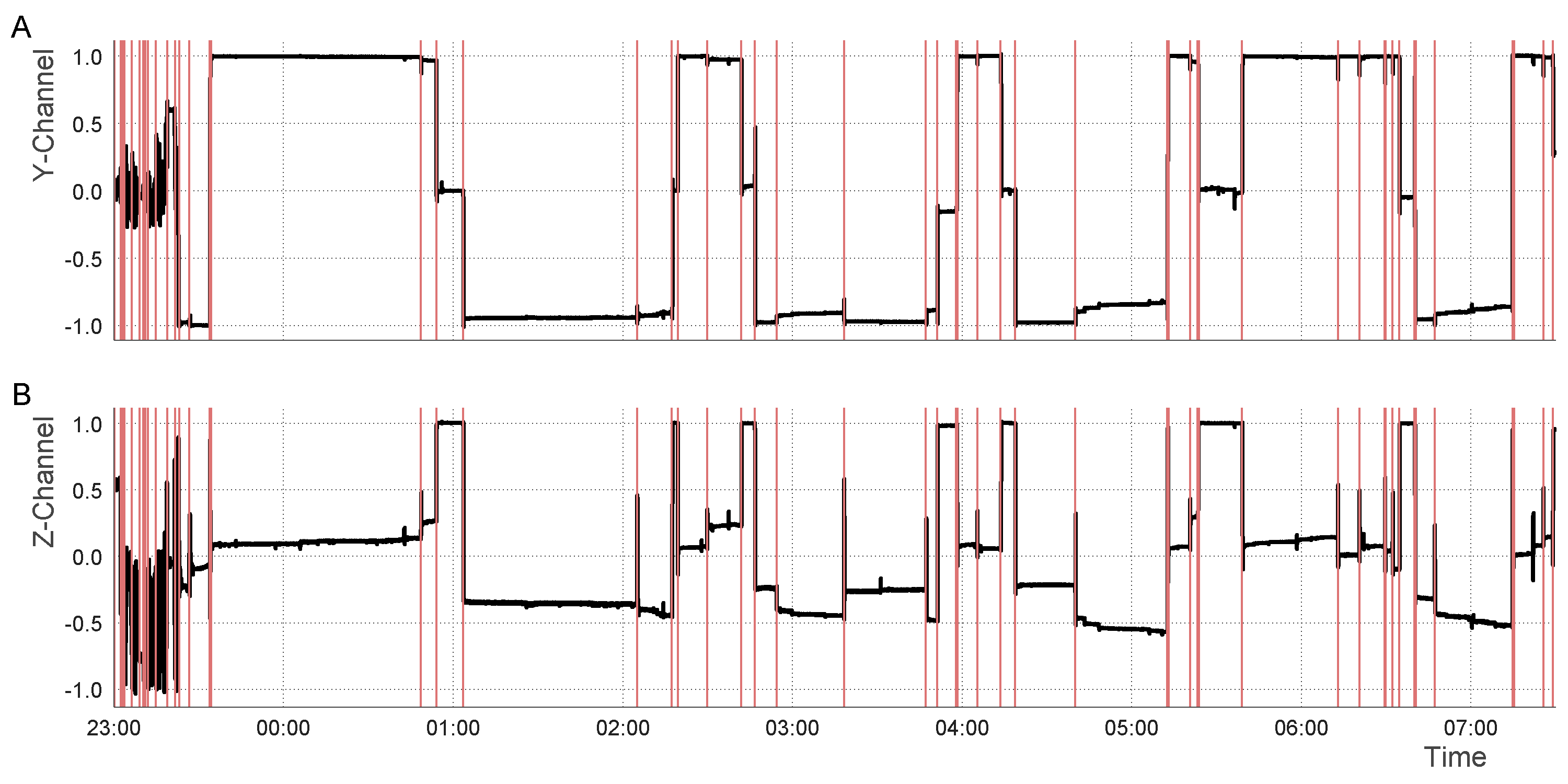


**Figure S1. Output from the stable posture identification algorithm. (A) and (B) are the y and z channels from chest actigraphy respectively. Time is taken over a period the participant reported begin in bed, in this case about 23:00-8:00. The vertical red lines indicate changes in posture.**

**Wrist movement results**


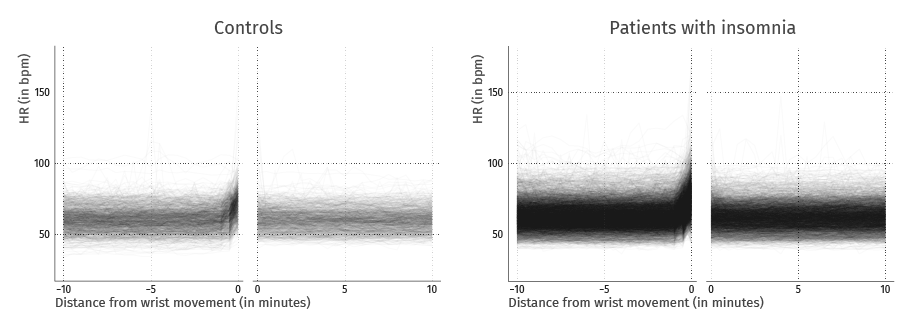


**Figure S2. Heart rate prior and after a nocturnal wrist movement.** Lines indicate individual trajectories to and from single movement episodes. The missing central part indicates movement episodes of differing length.

|  | **Insomnia** | **Controls** | **Group differences** | |
| --- | --- | --- | --- | --- |
| **Interval**  **(seconds)** | **HR average (bpm)** | **HR average (bpm)** | **HR average (bpm)** | ***p*** |
| 90–60 | 62.7  [61.34, 64.06] | 59.9  [57.16, 62.64] | 2.82  [-0.12, 5.76] | .060 |
| 60–30 | 62.9  [61.54, 64.26] | 60.1  [57.36, 62.84] | 2.76  [-0.18, 5.70] | .066 |
| 30–0 | 63.3  [61.94, 64.66] | 60.5  [57.76, 63.23] | 2.80  [-0.14, 5.74] | .062 |
| Epoch with movement | 66.1  [64.74, 67.46] | 62.9  [60.16, 65.64] | 3.13  [0.19, 6.07] | .038 |
|  |  |  |  |  |
|  | **Insomnia** | **Controls** | **Group differences** | |
| **Interval**  **(seconds)** | **HR change (bpm)** | **HR change (bpm)** | **HR change (bpm)** | ***p*** |
| 90–60 vs 60–30 | 0.14  [-0.16, 0.44] | 0.21  [-0.41, 0.84] | 0.06  [-0.21, 0.33] | .860 |
| 60–30 vs 30–0 | 0.38  [0.07, 0.68] | 0.34  [-0.28, 0.96] | -0.04  [-0.31, 0.23] | .912 |
| 30–0 vs Epoch with movement* | 2.81  [2.50, 3.11] | 2.48  [1.85, 3.11] | -0.33  [-0.60, -0.6] | .356 |

**Table S5. Pairwise comparisons of HR slope during subsequent epochs prior to wrist movements.** Asterisks following the interval contrast indicate that the change in HR significantly predicted impending movement.

|  | **Insomnia** | **Controls** | **Group differences** | |
| --- | --- | --- | --- | --- |
| **Interval**  **(seconds)** | **HR average (bpm)** | **HR average (bpm)** | **HR average (bpm)** | ***p*** |
| Movement | 62.0  [60.65, 63.34] | 59.6  [56.85, 62.35] | 2.34  [-0.62, 5.29] | .119 |
| 0–30 | 61.8  [60.45, 63.15] | 59.1  [56.35, 61.84] | 2.72  [-0.24, 5.68] | .071 |
| 30–60 | 61.5  [60.15, 62.84] | 59.0  [56.25, 61.75] | 2.54  [-0.42, 5.50] | .091 |
| 60–90 | 61.4  [60.05, 62.75] | 58.9  [56.15, 61.64] | 2.50  [-0.46, 5.46] | .094 |

**Table S6. Pairwise comparisons of HR slope during subsequent epochs after wrist movements.**

**24-hr HRV mixed model results**

|  |  | | β | *SE* | t | *p* |
| --- | --- | --- | --- | --- | --- | --- |
| **HR** | |  |  |  |  |  |
|  | Intercept | | 0.75 | 0.13 | 56.04 | <.001 |
|  | ISI_group | | 3.39 | 1.45 | 2.33 | .021 |
|  | State | | -0.15 | 0.05 | -288.39 | <.001 |
|  | Sex | | -4.80 | 1.21 | -3.96 | <.001 |
|  | Age | | -0.71 | 0.55 | -1.30 | .197 |
|  | ISI_group*state | | 0.385 | 0.06 | 6.64 | <.001 |
| **HFnu** | |  |  |  |  |  |
|  | Intercept | | 0.32 | 0.15 | 21.13 | <.001 |
|  | ISI_group | | -0.02 | 0.16 | -1.33 | .185 |
|  | State | | 0.09 | 0.00 | 65.34 | <.001 |
|  | Sex | | -0.09 | 0.01 | -6.44 | <.001 |
|  | Age | | -0.02 | 0.01 | -3.49 | <.001 |
|  | ISI_group*state | | 0.01 | 0.00 | 3.20 | .001 |
| **Sdnn** | | |  |  |  |  |
|  | Intercept | | 34.98 | 2.04 | 17.14 | <.001 |
|  | ISI_group | | -2.69 | 2.17 | -1.23 | .218 |
|  | State | | 5.76 | 0.15 | 38.88 | <.001 |
|  | Sex | | 7.41 | 1.81 | 4.09 | <.001 |
|  | Age | | -6.02 | 0.82 | -7.32 | <.001 |
|  | ISI_group*state | | -0.65 | 0.16 | -3.91 | <.001 |
| **Rmssd** | | |  |  |  |  |
|  | Intercept | | 31.94 | 2.48 | 12.86 | <.001 |
|  | ISI_group | | -5.17 | 2.64 | -1.95 | .052 |
|  | State | | 9.16 | 0.15 | 61.21 | <.001 |
|  | Sex | | 3.10 | 2.20 | 1.41 | .161 |
|  | Age | | -6.04 | 1.00 | -6.03 | <.001 |
|  | ISI_group*state | | -0.45 | 0.16 | -2.72 | .007 |

**Table S7.** Full linear mixed model results.
